# Genetic Associations of Angiotensin-Converting Enzyme with Primary Intracerebral Hemorrhage: A Meta-analysis

**DOI:** 10.1371/journal.pone.0067402

**Published:** 2013-06-27

**Authors:** Yuhao Sun, Ye Liu, Lora Talley Watts, Qingfang Sun, Zhihong Zhong, Guo-Yuan Yang, Liuguan Bian

**Affiliations:** 1 Department of Neurosurgery, Ruijin Hospital, Shanghai Jiao Tong University School of Medicine, Shanghai, China; 2 Department of Neurology, Ruijin Hospital, Shanghai Jiao Tong University School of Medicine, Shanghai, China; 3 Research Imaging Institute, University of Texas Health Science Center at San Antonio, San Antonio, Texas, United States of America; 4 Neuroscience and Neuroengineering Research Center, Med-X research Institute, Shanghai Jiao Tong University, Shanghai, China; University G. D'Annunzio, Italy

## Abstract

**Background:**

A number of studies have reported an association of angiotensin-converting enzyme (ACE) gene polymorphism with primary intracerebral hemorrhage (PICH), however the reports have demonstrated inconclusive results. To clarify this conflict, we updated the previously performed meta-analysis by Peck *et al.,* which revealed negative results, by investigating the ACE polymorphism and its correlation to PICH.

**Methods:**

PubMed and Embase databases (through Dec 2012) were searched for English articles on the relationship of the I/D polymorphism in ACE with PICH in humans. Summary odds ratios (ORs) were estimated and potential sources of heterogeneity and bias were explored.

**Results:**

A total of 805 PICH cases and 1641 control cases obtained from 8 case-control studies were included. The results suggest that in dominant genetic models, the ACE I/D polymorphic variant was associated with a 58% increase in susceptibility risk of PICH (OR = 1.58; 95% CI = 1.07–2.35 for DD vs. DI+II). However, in the subgroup analysis based on race, a significant increased risk was found in Asian DD homozygote carriers (OR = 1.76 and 95% CI = 1.16–2.66 for DD vs. DI+II), but not in Caucasian DD homozygote carriers (OR = 1.18, 95% CI = 0.36–3.88, P = 0.784 for DD vs. DI+II). The heterogeneity between studies was remarkable, and its major sources of heterogeneity were due to the year in which the study was published. No potential publication bias was observed in dominant genetic models.

**Conclusions:**

These data demonstrated evidence of a positive association between ACE I/D polymorphism with PICH, and suggested that the ACE gene is a PICH susceptible gene in Asian populations.

## Introduction

Primary intracerebral hemorrhage (PICH) represents about 15% of all occurring strokes, and has much higher mortality rate than ischemic stroke. In the United States approximately 20,000–40,000 new cases of PICH emerge each year [1], [2], with more than 50% of these patients resulting in death or severe disability, even when provided with best medical care [3]. Epidemiological studies have highlighted differences in the incidence of PICH between different ethnicities [4], with African Americans having twice the incidence rate and Japanese having four times the incidence rate than the Caucasian population [5]. Although hypertensive arteriolosclerosis and cerebral amyloid angiopathy account for the majority of sporadic PICH, a growing spectrum of genetic risk factors have more recently been identified. Characterization of these genetic variations may allow for improved prognostication and prevention of PICH.

Growing evidence implicates angiotensin–converting enzyme (ACE), a key enzyme of the rennin-angiotensin system, as an important modulator of cerebrovascular disease (CVD). The ACE gene is located on chromosome 17q23 and consists of 26 exons and 25 introns. A crucial polymorphism termed Ins/Del occurs due to an insertion (I) or deletion (D) of 287 base pairs within intron 16. The D allele of the polymorphism accounts for significantly higher serum ACE levels and activity, compared to carriers of the insertion allele [6]. Additionally, increased serum levels of ACE may contribute to vascular injury, which can further lead to CVD. There have been several reports on the association of the ACE gene polymorphism with atherosclerosis [7]. Moreover, ACE has been shown to inhibit atherosclerosis progression [8]. Although recent data lack significant evidence of neuroprotection in patients with ICH [9], these findings imply that the ACE gene may be related to a predisposition to ICH.

In view of few modifiable risk factors for PICH, acute therapeutic options are limited to controlling hypertension. Improving risk assessments through the identification of genetic variants in ICH may contribute to prevention efforts. A previous systematic review of hemorrhagic stroke, published in 2008, failed to confirm a significant and consistent association with ACE I/D [10], likely due to low statistical power and the small number of studies utilized in this study. Moreover, the evidence was controversial, and as a result scholars have become engaged to provide novel views on this issue recently.

Here, we conducted a comprehensive literature-based meta-analysis in which we updated the sample size and explored the ACE I/D variants association with PICH.

## Methods

### Data Sources and Searches

We independently searched papers using Medline (PubMed) and Embase through Dec 2012 to identify eligible genetic association studies evaluating ACE genotype and PICH. Search terms included hemorrhagic stroke, intracerebral hemorrhage and cerebral amyloid angiopathy, in combination with polymorphism or SNP. Search results were limited to articles of human studies written in English. All references cited in the identified publications were also searched for additional studies not indexed in these two databases. Case reports, editorials, and review articles were excluded.

### Study Selection and Eligibility Criteria

The inclusion criteria were: (1) case–control studies and cohorts which evaluated the association between ACE I/D polymorphism and PICH risk; (2) studies using validated molecular methods for genotyping; and (3) studies containing independent data of ACE I/D genotype frequency for estimating an odds ratio (OR) with a 95% confidence interval (CI). Cases were confirmed with diagnosis of intracerebral hemorrhage based on neuroimaging (magnetic resonance imaging or computerized tomography). Control populations: The vast majority of subjects classified as ‘controls’ were not subjected to neuroimaging, based on the lack of symptoms present following a clinical assessment for hemorrhage.

Studies were excluded if (1) the patients were children (age<18 years), (2) the genotype frequency was not adequately reported (and such data could not be obtained from the authors), (3) a case was caused by trauma, hemorrhagic rupture of a tumor or arteriovenous malformation, (4) the study focused solely on intracranial hemorrhage following acute ischemic stroke.

For studies containing overlapping cases or controls, the most recent and/or largest study with extractable data was included to avoid double counting.

### Data Extraction

From each selected study the following information was extracted: name of first author, journal, year of publication, country, ethnicity, demographic characteristics, number of ICH cases and controls, confirmation of diagnosis, genotyping method, genotype distributions and allele frequency, and whether genotypes were stated to be in Hardy-Weinberg equilibrium (HWE). If the frequencies of the alleles and/or the genotypic distributions were unavailable, detail information was calculated from the papers. We did not contact individual authors for further information.

The initial data extraction was undertaken by Y Liu. Several subsequent passes were then confirmed by Y Sun to ensure the comprehensive inclusion of all appropriate studies. All studies were adjudicated by 2 independent reviewers (Q Sun and Z Zhong), with disagreements resolved through discussion and consensus.

### Assessment of Study Quality

Two investigators (Y Liu and Y Sun) independently rated the quality of each retrieved study using a 10-point scoring system developed for this meta-analysis. The scoring system was based on factors that we believed would be indicators of high quality observational studies (Table 1). Study design, study size, source of population, genotyping method, genotypes stated to be in HWE and adjusted ORs were included in our evaluation of the quality of each assessed study. We assessed each study against a checklist of key quality indicators, however, studies were not included or excluded on the basis of these quality indicators.

**Table 1:** Summary of key quality indicators.

Reference#	Study design		No. of Cases/Study size (0–2)*	Mutation Detection		HWEstated (1)	Source of population			Adjusted resultsfor other riskfactors (1)	No. of quality indicators(out of 10)
	Cohort/Nestedcase-control (2)	Incidence/Prevalencecase-control (1)		DNASequencing (2)	ASO(1)		OneHospital (0)	Multi-Centre (1)	Community-based (2)		
Andrew C	√		193/698		√	√		√			7
Nakata Y		√	38/192		√	√		√			4
Lin JJ		√	92/606		√	√		√		√	6
Chowdhury AH		√	78/423		√	√	√			√	5
Slowik A		√	58/384		√	√	√				4
Li Y		√	81/468		√	√			√	√	7
Chen CM		√	217/500		√	√	√			√	6
Kalita J		√	183/574		√	√	-	-	-	√	6

Abbreviations: ASO, PCR-based allele-specific oligonucleotide; HWE, Hardy-Weinberg equilibrium.

#Sources are listed by publish year.

* n>100: score = 2; n = 50–100: score = 1; n<50: score = 0.

### Statistical Analysis

A list of details extracted from each study is included in Table 2. We performed a meta-analysis to investigate the association between ACE I/D and PICH for the allele contrast (D vs I), and the recessive (DD vs ID and II) and dominant (DD and ID vs II) models as previously utilized [11]. To measure the strength of association between ACE I/D polymorphism and ICH risk, overall odds ratios (OR), with the corresponding 95% confidence intervals (CI) were assessed. OR was calculated by a fixed-effects model using the Mantel-Haenszel method [12], [13] or the random-effects model using the DerSimonian and Laird method [14] according to the heterogeneity. Statistical heterogeneity across the various studies was determined by the Cochran’s X^2^ based Q statistic and I^2^ with statistical significant considered at P<0.10 [15]–[17]. When the P value was greater than 0.10, the fixed-effects model was used to pool the data, otherwise, a random-effects model was utilized. The significance of the pooled OR were determined by Z-test, with a P value less than 0.05 considered statistically significant. When we searched the HapMap database, we identified different linkage disequilibrium groups around the ACE gene among different ethnicities. Therefore, Caucasian and Asian participants were separated according to additional sensitivity analysis.

**Table 2:** The distribution of the ACE I/D variant for cases and controls.

Reference	Distribution of genotype						Distribution of allele			
	II		ID		DD		I		D	
	Case	Control	Case	Control	Case	Control	Case	Control	Case	Control
Andrew C	17	50	21	102	10	63	55	202	41	228
Nakata Y	18	11	17	22	3	5	53	44	23	32
Lin JJ	31	113	43	142	18	45	105	368	79	232
Chowdhury AH	32	79	37	97	9	14	101	255	55	125
Slowik A	10	27	22	57	26	32	42	111	74	121
Chen CM	85	159	108	101	24	23	278	419	156	147
Li Y	8	59	32	139	41	113	48	257	114	365
Kalita J	39	64	89	102	65	22	167	230	219	146

The Begg’s funnel plots and Egger’s test were conducted to statistically assess potential publication bias (using a p value of <0.05) [18]. All statistical tests were performed using STATA 10.0 software (StataCorp, College Station, TX, USA). We prepared this report in accordance to the Meta-analysis of Observational Studies in Epidemiology reporting guidelines [19].

## Results

### Search Results

**Figure 1:**
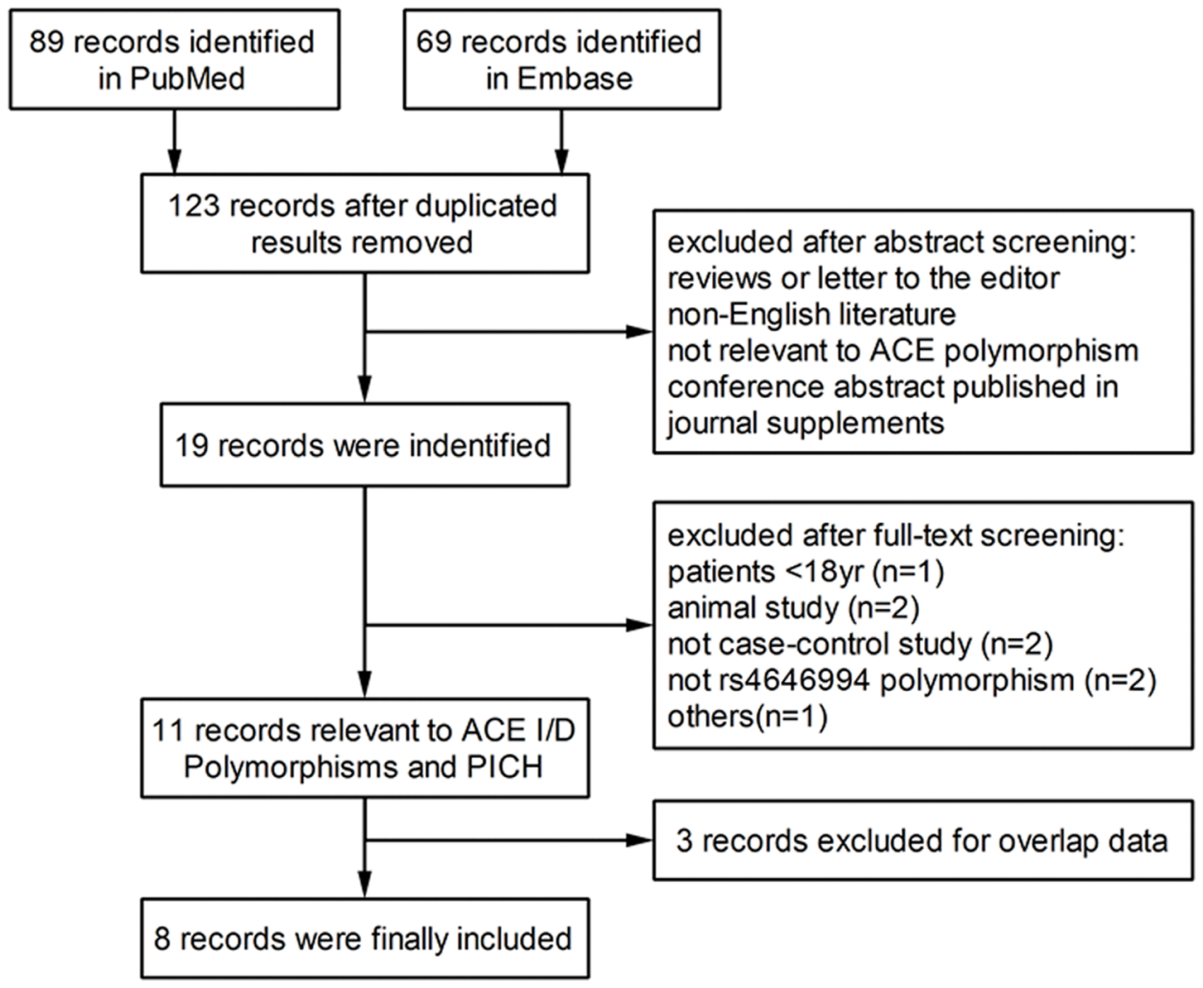
Study selection and exclusion. Flowchart of retrieved and excluded studies, with specification of the reasons.

A flow diagram of the performed database search is shown in Figure 1. Combining PubMed and Embase searches yielded 123 studies that were further screened using titles, abstracts, and keywords. According to the inclusion and exclusion criteria, we included 8 studies for meta-analysis of the association between ACE I/D polymorphism and PICH [20]–[27]. The complete data set included 1641 healthy people from the general population and 805 patients with primary intracerebral hemorrhages.

### Study Characteristics

The included studies were published from 1996 through 2011. All 8 studies evaluated susceptibility to PICH. The characteristics of each study are summarized in Table 3. The majority of studies were conducted on Asian populations and the remaining 2 studies were conducted on Caucasian populations. Studies had a mean of 480.6 (range 192 to 698) total participants. The mean age of patients diagnosed with PICH ranged from 55 to 74 years. 5 out of the 8 studies used frequency-matched controls based on gender and/or age. The larger studies generally fulfilled more of our quality indicators for PICH genetics than the smaller studies (table 1). The genotype distributions among the controls were in agreement with HWE for all included studies.

**Table 3:** Characteristics of included studies.

Year	Author	Country	Ethnicity	Cases with PICH			Control			Information of PICH	Information of controls	Match criteria
				N	Male%	Mean age	N	Male%	Mean age			
1996	Andrew C	UK	Caucasians	48	48.0	74	124	46.3	72.5	Inpatients from four hospitals in Leeds, and were part of a cohort study examining the role of genotypes and hemostatic factors in the development of CVD	Randomly selected from general practice registers free of significant vascular disease from the same geographic locality.	/
						(26–92)			(20–90)			
1997	Nakata Y	Japan	Asian	38	68.4	63±10	12	68.4	63±10	Cases from six hospitals in Osaka, Japan,aged from 30–80 yr	Individuals without a history of CVD or transient ischemic attack from onehospital undergoing general check-ups	Age, sex & history of hypertension
2000	Lin JJ	China	Asian	92	55.6	57.2±10.1	136	54.7	55.6±7.6	Inpatients from either hospitals in two regions of Taiwan	Healthy volunteers selected by cluster sampling from the same regions	/
						(42–84)			(44–92)			
2000*	Chowdhury AH	Bangladesh	Asian	45/	77.1/	49.8±8.5/	37/	67.0/	53.8±6.1/	patients admitted to the neurology and medicine units in Dhaka Medical College Hospital (DMCH), Dhaka	Idiopathic cataract patients (no historyof stroke) admitted in DMCH for elective surgery during the same period	/
				33	81.8	68.8±5.5	25	68.8	69.4±5.1			
2004	Slowik A	Poland	Caucasians	58	51.7	58.9±11.6	56	51.7	58.6±11.8	Admitted to the Department of Neurology, Jagiellonian University, Medical College	Free of any stroke	Age and Sex
2008	Chen CM	China	Asian	217	69.1	60.5±13.4	105	62.9	61.3±10.7	From Department of Neurology, ChangGung Memorial Hospital	Subjects came for a health exam or diseases other than CNS diseases	Age and Sex
2010	Li Y	China	Asian	81	0.0%	56.6±7.8	311	0.0%	56.9±8.1	Incident cases of married women born after June 1932 in 25 towns of two cities, who survived the episode of stroke	Admitted patients (or neighborhood)over the same period due to otherdiseases but not stroke or CVD	Age
2011	Kalita J	India	Asian	193	69.4	55	69	65.2	54.3±9.7/	Cases of PICH without trauma, tumor, vascular malformation and coagulopathy	Same ethnic healthy volunteers fromsame geographical region withoutneurological deficit and history of hypertension	Age and sex
						(16–65)			55.7±12			
									(F/M)			

* represent of 2 subgroup data of the same study (≤60yr/>60yr).

### Quantitative Data Synthesis

**Figure 2:**
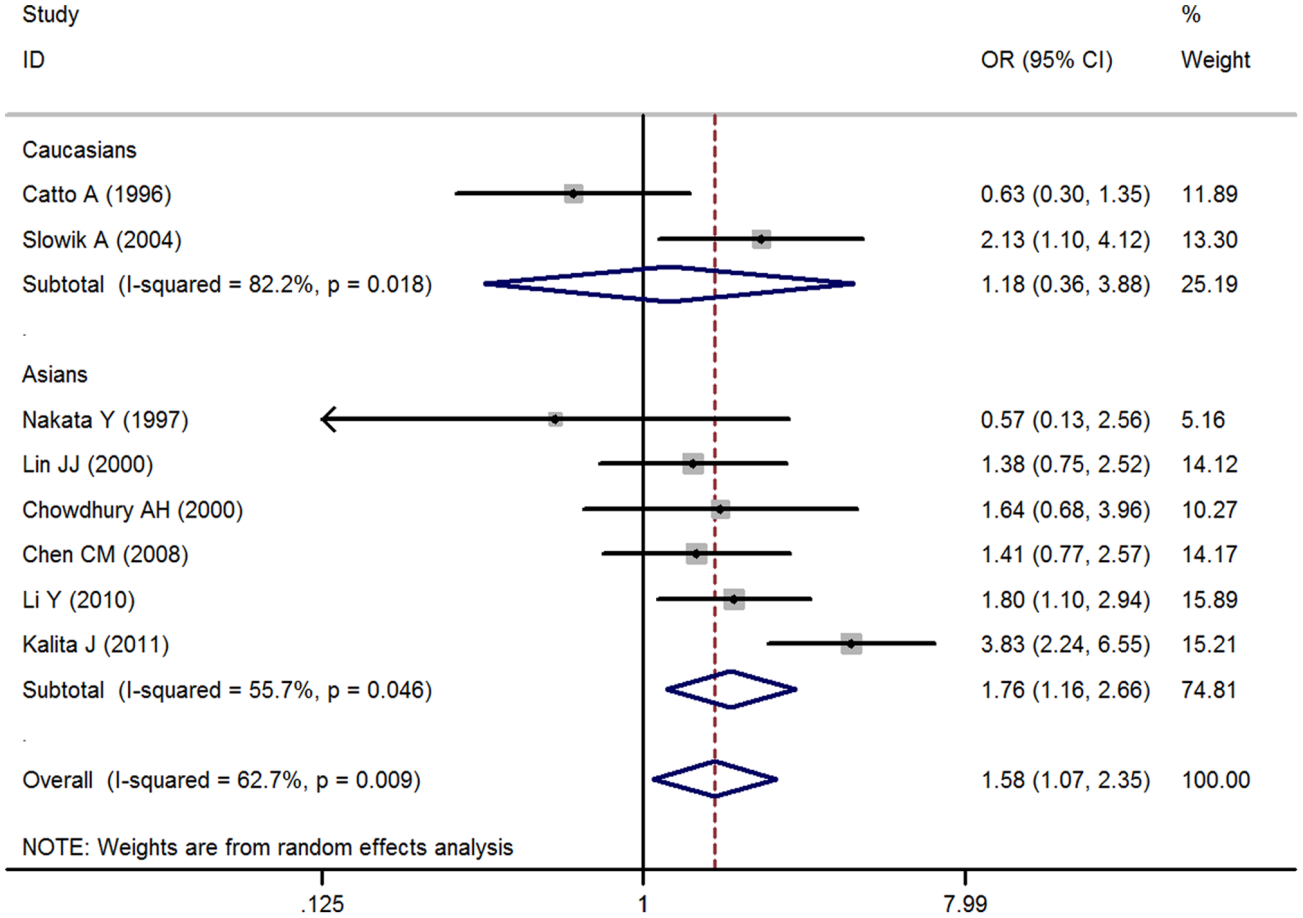
Meta-analysis with a random-effects model for the association between PICH risk and the ACE I/D polymorphism: subgroup analysis by race (dominant model).

Genotype and allele distributions for each study are shown in Table 2. In Figure 2, the heterogeneity of dominant, recessive model and allele model for all eight studies was analyzed. In Figure 2, the I-square value was 62.7% for the dominant model, 68.1% for the recessive model and 75.4% for allele contrast, respectively, suggesting moderate (50–75%) and high (>75%) heterogeneity. The main analysis for investigating the association between the DD genotype and the risk of PICH relative to the DI/II genotype revealed significant heterogeneity (P = 0.09) among the 8 studies using the dominant model. Additionally, the pooled random effects OR was significant (OR = 1.582 [95% CI, 1.067–2.347]). However, the recessive and allele models showed no significant association (random effects ORs, 1.254 [95% CI, 0.871–1.806] and 1.273 [95% CI, 0.972–1.667], respectively, Figures S1 & S2). The additive and codominant model produced a non-significant association (OR = 1.645 [95% CI, 0.961–2.816], OR = 0.900 [95% CI, 0.678–1.197]) as anticipated (figure not shown).

In addition, the Z value for the test for overall effect was 2.28 (P = 0.022) for DD vs. DI+II model, 1.22 (P = 0.224) for DD+DI vs. II model, 1.75 (P = 0.080) for D allele vs. I allele, suggesting that subjects who carry the DD genotype are more vulnerable to PICH compared to those in the dominant model. Overall, the results suggest that the variant genotypes of ACE I/D polymorphism were associated with a significantly higher risk of PICH when all the eligible studies were pooled into the meta-analysis.

### Subgroup Analyses

To control influence by ethnic heterogeneity, we separated the studies into those with either Caucasian or Asian samples. In the two studies using Caucasian subjects, the patient population was comprised of 106 PICH patients and 331 healthy controls, and we found significant heterogeneity among the ORs (P_dominant_ = 0.018, P_recessive_ = 0.070, P_allele_ = 0.006). The pooled OR from the two studies did not show a significant association for any genetic model (P_dominant_ = 0.784, P_recessive_ = 0.780, P_allele_ = 0.945, Figures 2, S1 & S2). In the 6 Asian studies, comprised of 699 PICH patients and 1310 healthy controls, there was remarkable heterogeneity among the ORs. Asian subjects showed obvious increased risk under a dominant, recessive and allele model with a magnitude of effects similar to that of the main analysis. (P_dominant_ = 0.008, P_recessive_ = 0.070, P_allele_ = 0.020, Figures 2, S1 & S2).

### Study Quality

The meta regression in the dominant model demonstrated that the major source of heterogeneity was the publication year (P = 0.032). Although ethnic background produced a significant association, the ethnic effect in the meta regression was not significant (P = 0.483). For the gender difference, we found more males than females were included in the meta-analysis for both PICH patients and controls. There was no significant effect of gender in the meta regression analysis (P = 0.958).

### Sensitivity Analysis

To assess the stability of the results of the current meta-analysis, we performed sensitivity analysis by sequentially excluding each study. The pooled ORs ranged from 1.40 to 1.82 in the dominant model, and statistically similar results were obtained after sequentially excluding each study except for the Slowik’s or Li’s study. The pooled ORs ranged from 1.14–1.42 in the recessive model, and statistically similar results were obtained after sequentially excluding each study except for the Catto study. The pooled ORs ranged from 1.18–1.41 in the allele contrast model, and statistically similar results were obtained after sequentially excluding each study except for the Catto or Nakata studies. These results suggested that the stability of our meta-analysis is affected by these four studies.

### Publication Bias

**Figure 3:**
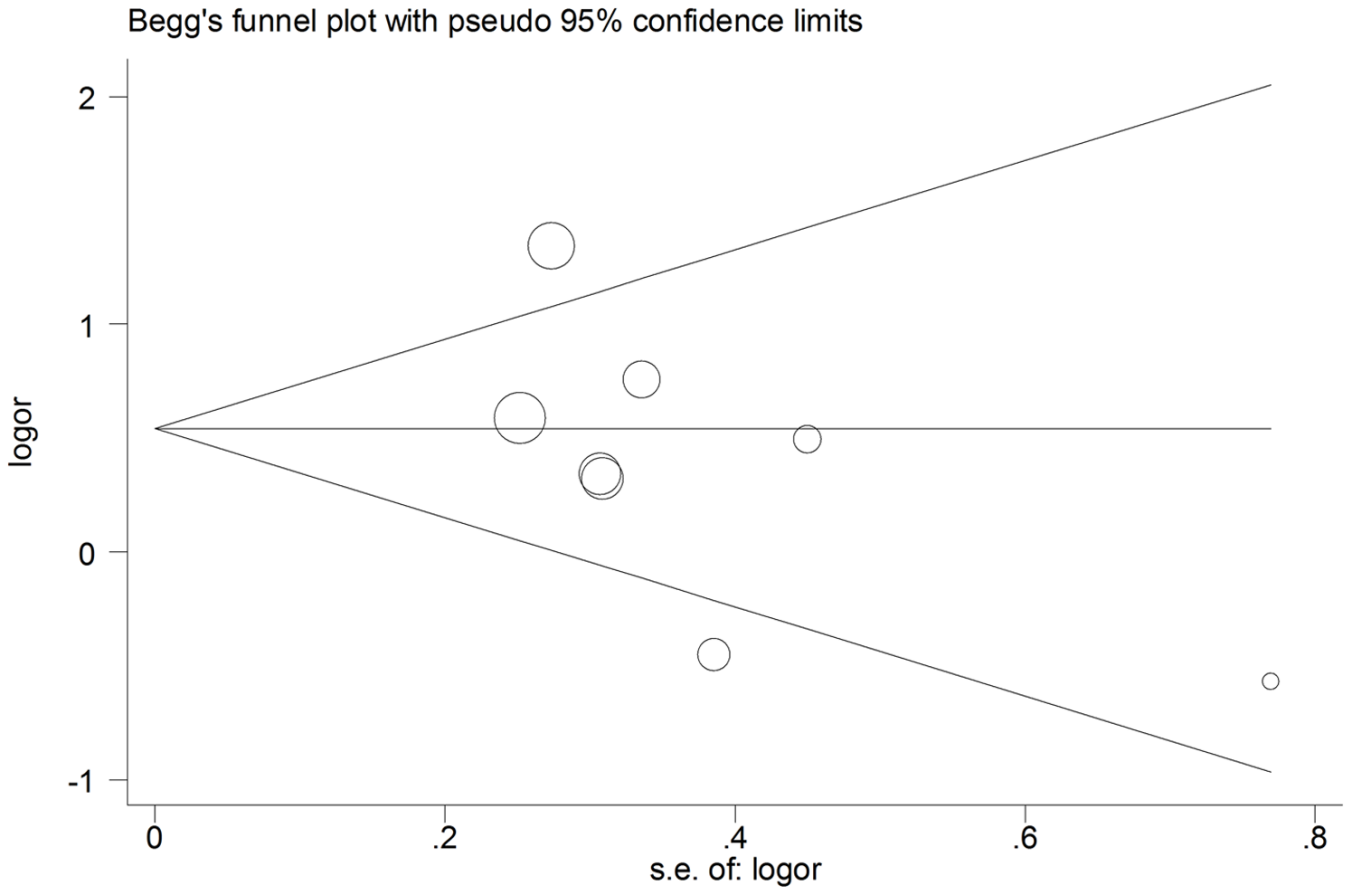
Begg’s funnel plot for publication bias in selection of studies of the ACE I/D polymorphism (dominant model).

Publication bias was assessed by the Begg’s funnel plot and Egger’s test. Visual inspection of the shape of the funnel plots showed symmetry in the DD vs. II+DI comparison genetic model, suggesting the absence of publication bias (Figure 3). Subsequently, the Egger’s test was performed to provide statistical evidence of the funnel plot asymmetry. The results indicated the current meta-analysis demonstrated a lack of publication bias using the dominant and recessive models, but did exist using the allele model (t = −0.160, P = 0.160 for DD vs DI/II; t = −1.78, P = 0.125 for DD/DI vs II; t = −2.49 P = 0.047 for D vs I, Figures S3 & S4).

## Discussion

The renin angiotensin system has been implicated in the pathophysiology of PICH by accelerating atherosclerosis [28]. Inactive angiotensin I can be transformed into vasoactive and idosterone-stimulating peptide angiotensin II through ACE, the key enzyme of the RAS [29]. A previous meta-analysis failed to detect an association between the ACE I/D polymorphism and PICH susceptibility [10]. The present meta-analysis explored a much larger sample size, and suggested a positive association between the ACE I/D polymorphism and PICH risk.

The use of composite quality scales may produce inherent problems and make it difficult to interpret the results, however the scale seemed to provide an overall assessment when comparing studies [30]. PICH is common in the aging population since the incidence of PICH increases gradually with age [31]. Previous reviews of sex differences in stroke epidemiology suggest that the incidence rates of intracerebral hemorrhage are higher among men [32]. Hence, if information of these risk factors were limited and could not be determined from the original studies, the association between ACE mutations and PICH risk could not to be congruently explained in the risk-unmatched, low-quality studies, compared to the age and sex-matched, high-quality studies. Given that heterogeneity comes from variations in study quality to a great extent [33]–[35] we should mention that significant heterogeneity existed in the overall comparison among the different genetic models. Ideally, age, sex and stable/exacerbation situation of hypertension should be matched in all cases and controls.

Considering that the role of ACE in the pathogenesis of cerebrovascular lesions remains unclear, our meta-analysis findings may account for the relationship of ACE polymorphism with PICH. The ACE D allele has been described to be associated with 28–47% of the variance of ACE activity both in the circulation [6], [36] and in tissues [37]. Increased levels of angiotensin II, converted by ACE, results in early arteriolar proliferation of smooth muscle [38],followed by smooth muscle cell death and collagen deposition [39]. This process impairs vasoconstriction and results in hypertension, which worsens arteriosclerosis, eventually leading to possible intracerebral hemorrhage. Moreover, chronic exposure to high levels of plasma ACE may contribute to increased vascular wall thickness and stiffness, which increases cerebrovascular risk as well [40]. These factors indicate that the genotype of the ACE I/D polymorphism are associated with higher PICH risk.

Subgroup analysis revealed a significant increased risk of PICH among Asians, but not in Caucasians, suggesting that ethnic differences in genetic backgrounds and the environment they lived in may be involve in the incidence of PICH. There are a number of factors that might explain the differences seen in the two populations studied. First, genetic backgrounds (SNP frequency) vary greatly from Asians to Caucasians. Second, the difference in living environments between these two populations may account for the different genetic effects. Thus, additional studies using different population are warranted to further validate ethnic differences on the impact of this polymorphism on PICH risk.

Only one study on women identified a positive association, therefore there was not enough power to investigate a potential gender interaction for the ACE I/D variant. Such a sex-specific influence may attribute to corticosteroid hormones, which play a crucial role in the renin-angiotensin-aldosterone system in a variety of tissues [41]. In future studies, more concentration on sex-dependent models should be taken into consideration to provide a more powerful analytical framework [42].

A potential publication bias may be present in the allele genetic model. Nevertheless, the combined risk estimate may not be changed just by modifying this bias using the trim and fill method. Though no significant publication bias for DD vs DI/II was found using the Begg’s funnel plots or the Egger’s test, the results should be explained with caution. Interpreting the p value in the setting of significant heterogeneity between studies may be problematic and limited.

There is a known positive association between hypertension and ICH [31]. Therefore, it is vital to know the HBP status of both PICH patients and controls. Questions like whether the patients were under ACE inhibitor usage or not should be known as well. We failed to analyze these issues due to lack of original data in this meta-analysis. Moreover, the combination of the polymorphisms and gene-environment interactions should be investigated. Despite difficulties in study design and the assessment of environmental factors, future case-control studies may help to resolve these questions if the studies include information on the status of hypertension, the use of ACE inhibitors and environmental exposures, such as smoking history. Patients who are at risk of mortality from PICH, as determined by ACE I/D polymorphism screening, may be chosen as good candidates for treatment with ACE inhibitors. However, an association between ACE I/D polymorphism and PICH mortality was not investigated as insufficient information (based on long term follow up for each patient) could not be universally provided by these studies.

Some limitations have already been considered when explaining the results in this meta-analysis. First, our study is under-powered to some degree due to its small sample size [43], and a genuine association between the ACE I/D polymorphism genotype and PICH susceptibility may fail to be detected due to the limited size. At the same time, it is difficult to reduce the rate of false discovery in all circumstances unless there is great similarity between study heterogeneity [44]. Therefore, more studies should be reviewed to obtain conclusive results. Second, the majority of the studies examined herein were from Asian populations, with only two of the studies examined from Caucasian populations, which may bias our results to be applicable only in Asian populations. Further population studies are required to investigate the association between the ACE I/D polymorphism and PICH risk in other races including African Americans and Latinos. Finally, the data were not subdivided into additional groups based on other variables, such as gender, due to the limitation of the original information for each patient. A more precise analysis could be performed if adjusted estimates were available in all studies.

### Conclusion

In conclusion, the present study provides the most comprehensive meta-analysis to date on the assessment of the relationship between the ACE I/D polymorphism and PICH risk. The present study supports an association between ACE I/D polymorphic variant and PICH in Asians, but not in Caucasians. However, additional large-scale studies with well-matched controls and multi-ethnic populations are required to validate our conclusions.

## Supporting Information

Figure S1 **Forest plot of OR with 95% CI for ACE I/D polymorphism in PICH susceptibility. (recessive model).**
(TIF)

Figure S2 **Forest plot of OR with 95% CI for ACE I/D polymorphism in PICH susceptibility. (allele model).**
(TIF)

Figure S3 **Begg’s funnel plot for publication bias in selection of studies of the ACE I/D polymorphism. (recessive model).**
(TIF)

Figure S4 **Begg’s funnel plot for publication bias in selection of studies of the ACE I/D polymorphism. (allele model).**
(TIF)
