# Correction: Genetic Associations of Angiotensin-Converting Enzyme with Primary Intracerebral Hemorrhage: A Meta-analysis

**DOI:** 10.1371/annotation/7f2d85e0-c41a-40cf-80e1-6ce4b0470eff

**Published:** 2014-01-06

**Authors:** Yuhao Sun, Ye Liu, Lora Talley Watts, Qingfang Sun, Zhihong Zhong, Guo-Yuan Yang, Liuguan Bian

The first and second authors, Yu-Hao Sun and Ye Liu, should be noted as having contributed equally to the article.

