# Supplementary figures and images for: Genetic Associations of Angiotensin-Converting Enzyme with Primary Intracerebral Hemorrhage: A Meta-analysis
Source: PLoS One. 2013 Jun 27;8(6):e67402. doi: 10.1371/journal.pone.0067402 (PMC3694901; doi:10.1371/journal.pone.0067402)

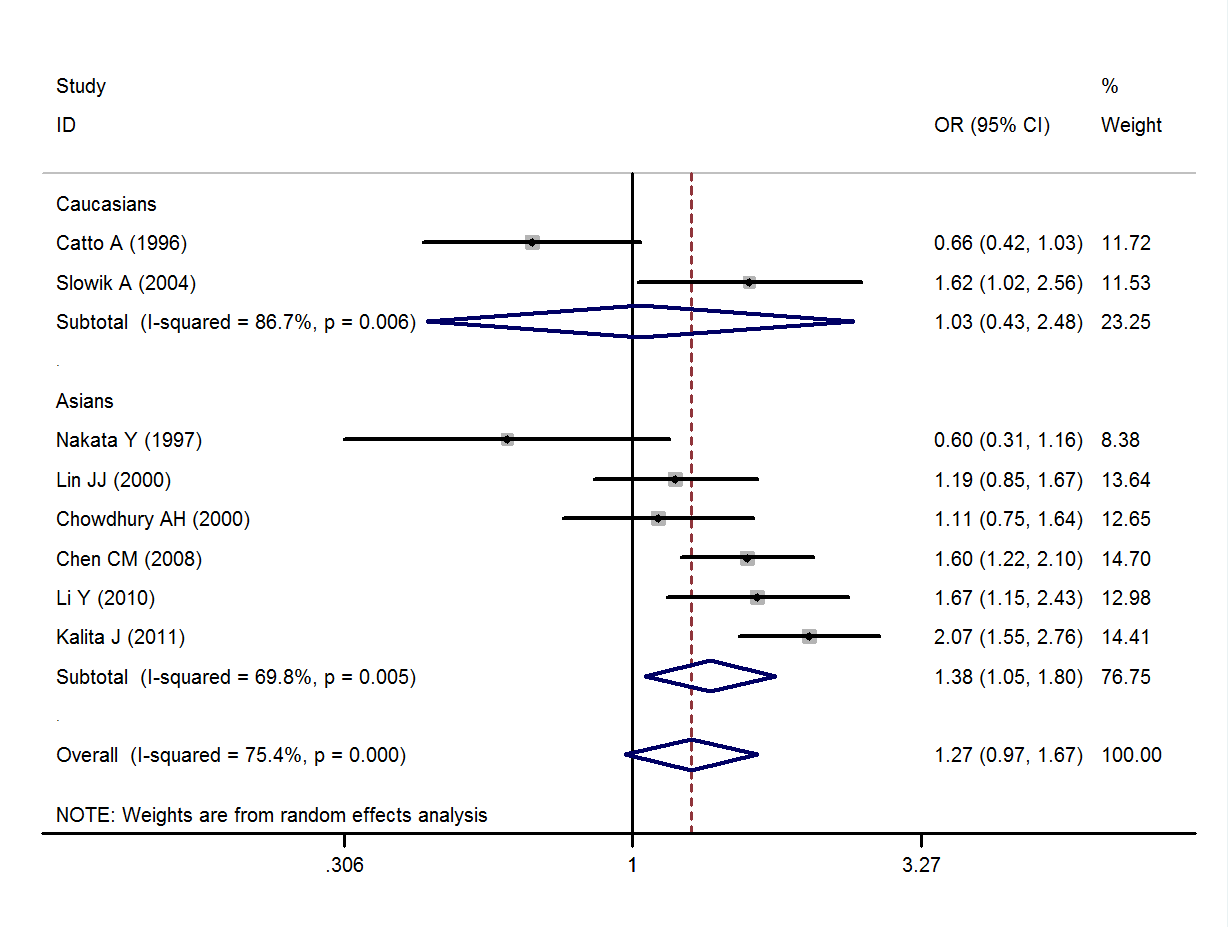

Supplement: Figure S1 — Forest plot of OR with 95% CI for ACE I/D polymorphism in PICH susceptibility. (recessive model). (TIF) [file pone.0067402.s001.tif]

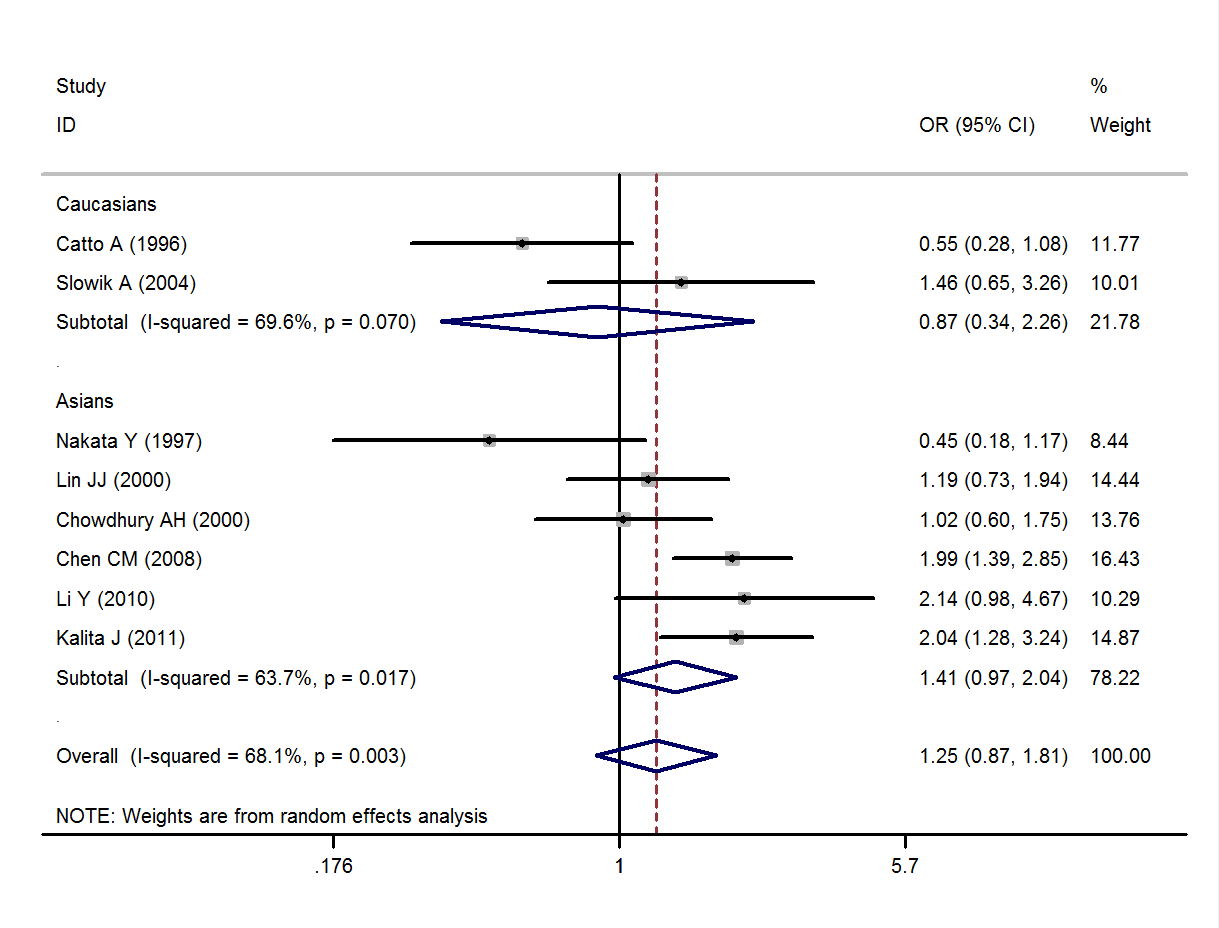

Supplement: Figure S2 — Forest plot of OR with 95% CI for ACE I/D polymorphism in PICH susceptibility. (allele model). (TIF) [file pone.0067402.s002.tif]

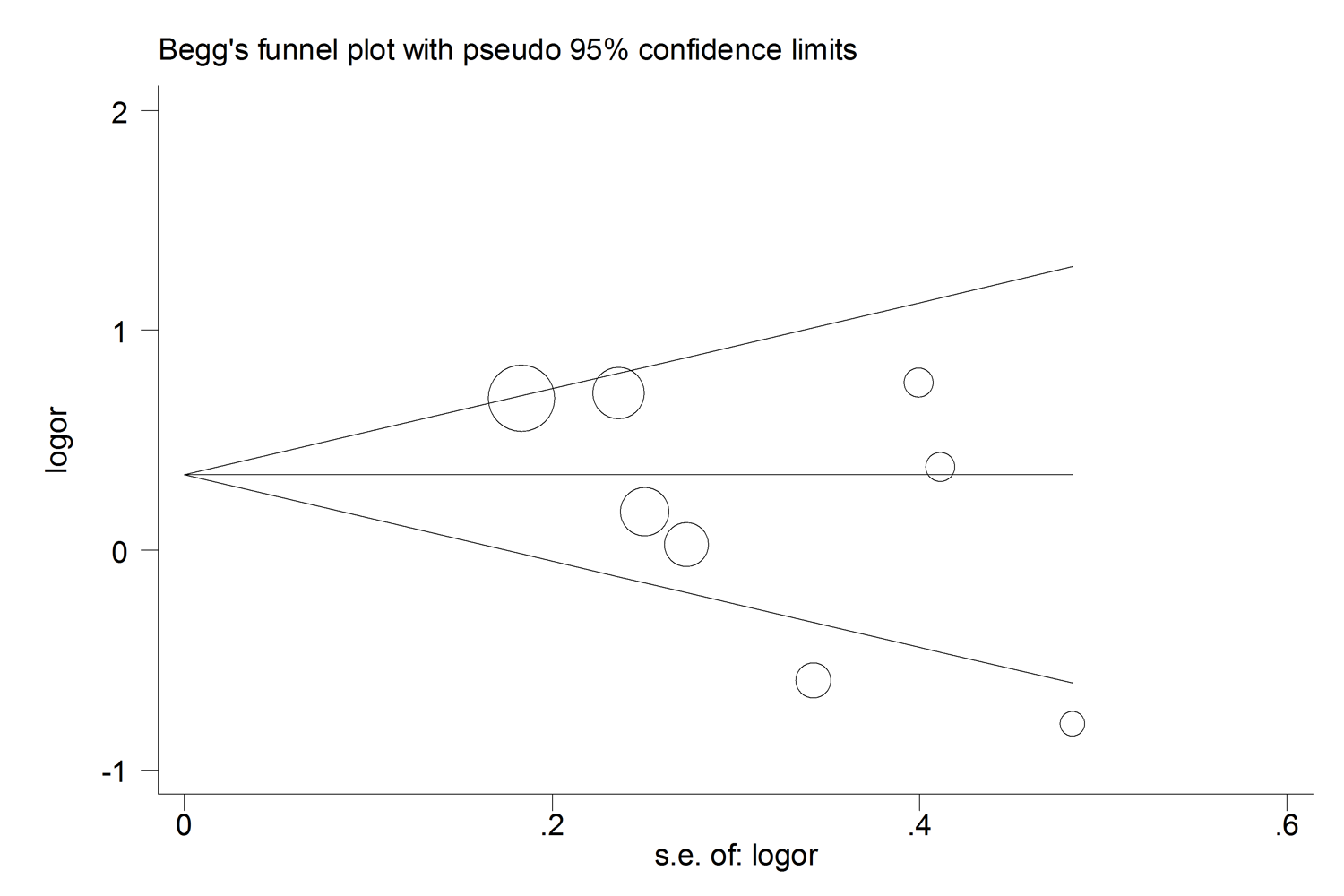

Supplement: Figure S3 — Begg’s funnel plot for publication bias in selection of studies of the ACE I/D polymorphism. (recessive model). (TIF) [file pone.0067402.s003.tif]

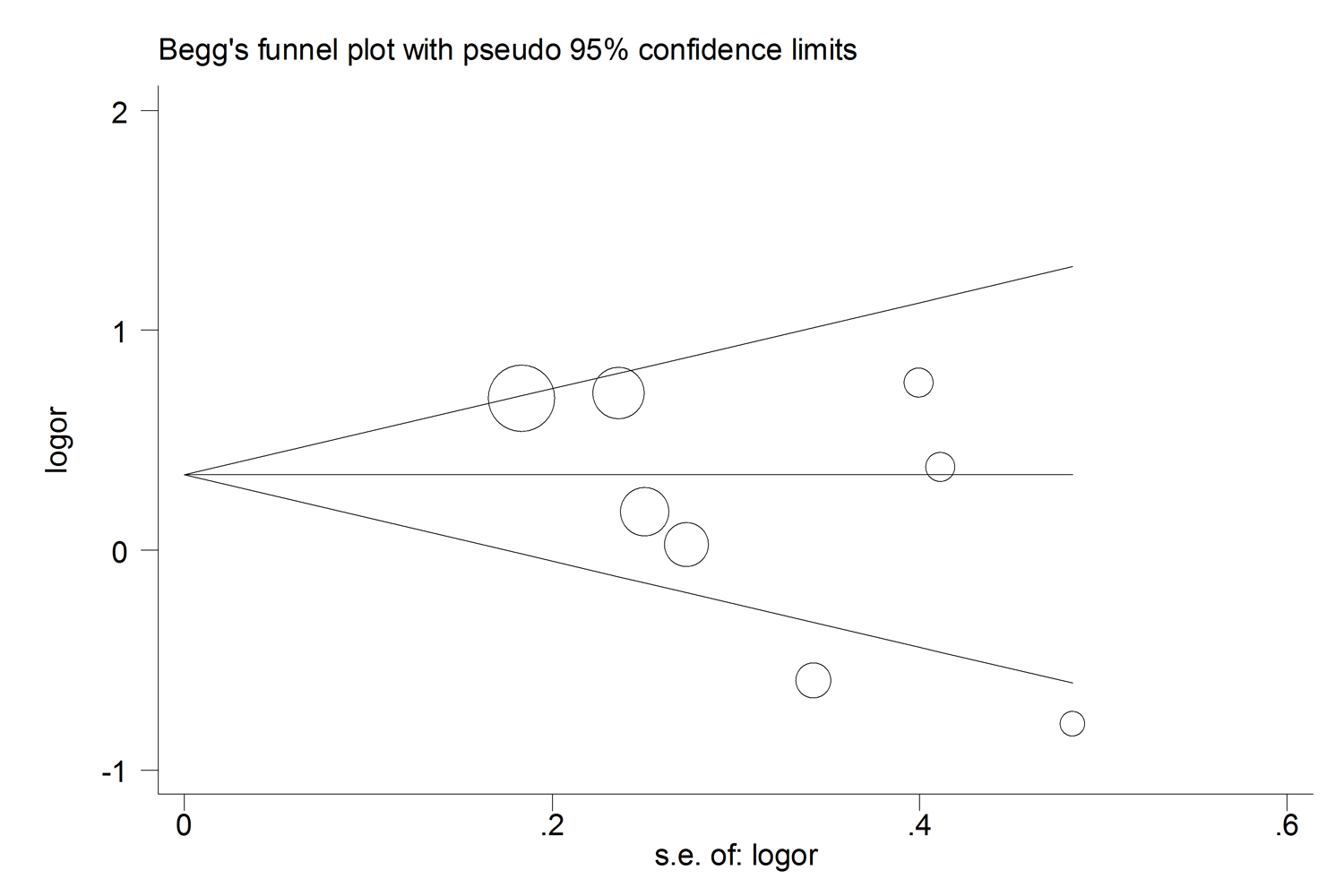

Supplement: Figure S4 — Begg’s funnel plot for publication bias in selection of studies of the ACE I/D polymorphism. (allele model). (TIF) [file pone.0067402.s004.tif]
